# Comparative Study of Fuzzy Rule-Based Classifiers for Medical Applications

**DOI:** 10.3390/s23020992

**Published:** 2023-01-15

**Authors:** Anna Czmil

**Affiliations:** The Faculty of Electrical and Computer Engineering, Rzeszow University of Technology, Powstancow Warszawy 12, 35-959 Rzeszow, Poland; czmilanna@gmail.com

**Keywords:** fuzzy rule-based system, interpretability, clinical decision support, medical diagnostic systems

## Abstract

The use of machine learning in medical decision support systems can improve diagnostic accuracy and objectivity for clinical experts. In this study, we conducted a comparison of 16 different fuzzy rule-based algorithms applied to 12 medical datasets and real-world data. The results of this comparison showed that the best performing algorithms in terms of average results of Matthews correlation coefficient (MCC), area under the curve (AUC), and accuracy (ACC) was a classifier based on fuzzy logic and gene expression programming (GPR), repeated incremental pruning to produce error reduction (Ripper), and ordered incremental genetic algorithm (OIGA), respectively. We also analyzed the number and size of the rules generated by each algorithm and provided examples to objectively evaluate the utility of each algorithm in clinical decision support. The shortest and most interpretable rules were generated by 1R, GPR, and C45Rules-C. Our research suggests that GPR is capable of generating concise and interpretable rules while maintaining good classification performance, and it may be a valuable algorithm for generating rules from medical data.

## 1. Introduction

Accurate diagnosis of patients with various illnesses and diseases is a challenging area of medical research. The key is predicting an outbreak of a disease, preventing the progression of chronic disease and saving lives if patients receive medical treatment immediately after diagnosis [1]. However, even the most experienced physician can become confused when a disease has several symptoms similar to another condition. A patient may also have a set of symptoms that can indicate various diseases, and these symptoms may not be easily quantifiable. When these symptoms occur, physicians at different professional and clinical levels can differ in their diagnosis, potentially resulting in a misdiagnosis. Moreover, patients are often uncertain of their symptoms, making the diagnosis more difficult. Therefore, computers have become crucial for medical diagnosis and prognosis in providing consistent results, especially with the growing amount of medical information [2]. However, machines cannot fully replace expert knowledge. Combining human expertise and computational models for advanced data analysis helps narrow the gap between acquiring and understanding data, which is vital for medical research. Experts need tools to transform raw and complex data into easily interpretable information, but the output of the algorithm alone is not sufficient for making an accurate diagnosis; expert knowledge is also required [3]. As diagnostic decision-making becomes more complex, developing highly effective and reliable medical decision support systems (MDSS) to support the complex and evolving diagnostic process is challenging [1].

Although data analytics for healthcare is gaining recognition rapidly, there are still limitations associated with machine learning algorithms that are black boxes. These algorithms contain a complex mathematical function, e.g., support vector machines (SVMs), or require an understanding of the distance function and the representation space, e.g., k-nearest neighbors (KNN), which are very challenging to explain and to be understood by experts in practical applications. However, the application of black-box algorithms in medicine has raised concerns in the academic community due to their opacity and lack of trustworthiness [4]. To summarize the performance of a model, it is necessary to report several metrics, since no single metric captures all the desired properties. Nevertheless, tools such as CACP simplify this task by allowing the assessment of classification efficiency, reproducibility, and statistical reliability while maintaining the objectivity of model comparisons [5].

Classification quality is crucial, but it is also essential to understand how a record is classified. MDSS rely on knowledge management to obtain clinical advice based on multiple factors in patient-related data. In these applications, models based on patterns, rules, or decision trees are more useful and easier for experts to comprehend in practical applications. In particular, rule-based systems (RBS) represent knowledge in the form of a set of rules that suggest what to do in various situations. They consist of a set of “if-then” rules, a set of facts, and interpreters that control the application of the rules. The idea of an expert system is to use the experience and facts in a knowledge base and encode it into a set of rules. If the expert system has access to the same data, it will behave similarly to the expert. RBS are straightforward models that can be adapted and applied to numerous problems [6]. Rule-based systems are known as white-box models because they provide a model closer to human language, making them easy for experts to understand [7]. The interpretability of a classification model is particularly important for MDSS. When designing classifiers, it is crucial to reach a compromise between interpretability and accuracy [8]. Accuracy is a well-known method for validating machine learning models in classification problems due to its popularity and relative simplicity. However, there is no widely accepted measure of the interpretability of machine learning models [9]. As it depends on several factors, mainly the structure of the model, the shape of the membership functions, the number of rules, attributes, and linguistic terms, it can be difficult to measure [8].

After introducing fuzzy rule-based systems (FRBS), which are models based on fuzzy sets proposed by Zadeh [10], many of their applications emerged in different areas such as artificial intelligence, robotics, decision-making, expert systems, power engineering, and medicine [11,12,13,14]. A fuzzy logic approach is an effective way to represent and understand data containing both patient information and clinical reasoning used by physicians to conclude patients’ health that is inherently uncertain and vague in medical problems. It has proven to be a powerful tool in developing decision support systems, such as rule-based medical decision support systems [3]. Many algorithms have been proposed for designing FRBS, including one rule (1R), C4.5 and its extensions, the exemplar-aided constructor of hyperrectangle (EACH), and repeated incremental pruning to produce error reduction (Ripper). 1R is a simple algorithm that uses a single rule to make predictions [15]. C4.5 and its extensions are decision tree learning algorithms that use fuzzy logic to make decisions at each node of the tree [16]. EACH is a clustering algorithm that uses fuzzy logic to group data into clusters [17], and Ripper is an algorithm that uses fuzzy logic to prune, or remove, unnecessary rules from a fuzzy rule-based system [18]. Genetic algorithms have been successfully applied to the generation of fuzzy rules and the adjustment of the membership functions of fuzzy sets [19]. Examples of these algorithms include hybrid decision tree-genetic algorithm (DT_GA), which combines a decision tree learning algorithm with a genetic algorithm [20], and the oblique decision tree with evolutionary learning (DT_Oblique), which uses evolutionary learning to improve the performance of an oblique decision tree [21]. Other examples include structural learning algorithm in a vague environment (SLAVEv0) and its extensions, which use genetic algorithms to learn the structure of a fuzzy rule-based system [22], the classifier based on fuzzy logic and gene expression programming (GPR) that combines fuzzy logic with gene expression programming to generate fuzzy rules for classification tasks [8], and hierarchical decision rules (Hider), which use genetic algorithms to generate fuzzy rules for classification tasks [23]. Organizational co-evolutionary algorithm for classification (OCEC) is another example of a genetic algorithm applied to fuzzy rule-based systems. This algorithm uses co-evolutionary learning, in which multiple populations of solutions are evolved simultaneously, to improve the performance of a fuzzy classifier [24]. Ordered incremental genetic algorithm (OIGA) [25] and Pittsburgh genetic interval rule learning algorithm (PGIRLA) [26] are both examples of genetic algorithms that are specifically designed for learning fuzzy rules. These algorithms use genetic operations to generate and refine a set of fuzzy rules that can be used to make decisions.

## 2. Related Work

Fuzzy logic is used extensively for medical applications by researchers for diagnosis and classification. For example, Aamir et al. used a fuzzy rule-based algorithm to predict the severity of diabetes in patients [27]. Adeli and Neshat found that a fuzzy rule-based algorithm was effective in diagnosing heart disease from electrocardiogram (ECG) data [28]. Improta et al. utilized a fuzzy rule-based algorithm for the evaluation of renal function in posttransplant patients [29]. Rotshtein proposed an approach for building a fuzzy expert system for the differential diagnosis of ischemia heart disease [30]. Mohammadpour et al. determined the accuracy of fuzzy rule-based classification that could non-invasively predict CAD based on the myocardial perfusion scan test and clinical-epidemiological variables [31]. Al-Dmour et al. presented the usage of fuzzy logic techniques in a warning system to categorize patients’ status or medical conditions [32]. RBS and FRBS have also been used to develop many MDSS in recent decades [31,33,34,35,36,37,38,39,40,41,42,43,44,45,46]. These systems represent the symptoms of MDSS patients and are based on an inference algorithm to process the information using linguistic terms. Domain knowledge is embedded as rules in the knowledge base.

Many studies demonstrate the potential of using different fuzzy rule-based algorithms in medical applications while simultaneously comparing different fuzzy algorithms. Steimann investigated the impact of fuzzy set theory on medical artificial intelligence and pointed out its most appreciated features [47]. Gupta et al. reviewed various fuzzy models that are being used in healthcare systems for making decisions. Mousavi et al. proposed an intelligent classification algorithm using a fuzzy rule-based approach to classify medical datasets and compared it with selected fuzzy rule-based algorithms [48]. Kluska and Madera proposed a new design for a very simple data-driven binary classifier and conducted an empirical study of its performance using other state-of-the-art algorithms and datasets from multiple disciplines, including medicine [8]. There are also many reviews in the literature on various fuzzy rule-based systems [49,50,51,52]. These works highlight important contributions, current trends, and challenges in the field.

Among the different reviews in the literature, choosing the type of fuzzy rule-based algorithm for particular medical applications remains a challenging task. The comparison of available algorithms is not straightforward, as researchers use various datasets and criteria for their evaluations. Another challenge is selecting an appropriate metric to evaluate the calculated results. Available research has not yet comprehensively investigated the validity of the outcomes of fuzzy rule-based algorithms using a wide range of available algorithms and metrics. Therefore, this study has two main objectives. First, we compare all commonly used, state-of-the-art algorithms and assess their performance. The comparison is made against the results of all selected algorithms compared in every dataset, calculated using 10-fold cross-validation. Our findings demonstrate a ranking of the algorithms in terms of the most popular performance metrics. Second, we analyze fuzzy rule-based classifiers in terms of rules’ size metrics and provide examples of rules generated by every algorithm to objectively determine which of these algorithms is worth using when applied to issues in clinical decision support. The use of some of those algorithms in the field of medicine is novel.

The remainder of the paper is structured as follows. Section 3 provides the details of the experimental datasets. Section 4 describes the applied fuzzy rule-based classification algorithms and their settings. Section 5 presents the classification assessment methods. Then in Section 6, the experimental results of the comparison are presented. Finally, Section 7 contains a discussion, observations, and conclusions.

## 3. Experimental Datasets

This article focuses on the medical applications of fuzzy rule-based classifiers, so only medical data are considered. Datasets were downloaded from the KEEL—dataset repository [53], and actual medical data were collected during other scientific research, as detailed below. We used standard classification datasets without missing values. Each dataset defines a supervised classification problem, and each example has some nominal and numerical attributes and a nominal output attribute. The datasets have different levels of class imbalance. Table 1 presents a summary of the datasets, including the number of records, attributes, classes, and class imbalance.

### 3.1. Appendicitis

The dataset includes 7 medical measures taken from 106 patients, along with a class label that indicates whether the patient has appendicitis (label 1) or not (label 0) according to the research by S. M. Weiss and C. A. Kulikowski [54].

### 3.2. Breast Cancer

The dataset of 277 instances with no missing values is characterized by 9 attributes provided by the Institute of Ljubljana Oncology. These attributes include both linear and nominal values, e.g., age, tumor nodes, and tumor size.

### 3.3. Haberman

The dataset contains 306 records described by 3 attributes of a study on the survival of patients who had undergone breast cancer surgery at Billings Hospital at the University of Chicago between 1958 and 1970. The task is to predict whether the patient survived for five years or more after surgery (positive) or died within five years (negative).

### 3.4. Heart

The heart disease database includes 270 instances with 13 attributes, each labeled with a class label indicating the absence (1) or presence (2) of heart disease. This dataset can be used to analyze various factors and characteristics that may be associated with heart disease.

### 3.5. Hepatitis

The dataset contains information on 80 patients affected by hepatitis, including a mixture of 19 integer and real-valued attributes. The task is to predict whether these patients will die (1) or survive (2).

### 3.6. Mammographic

The dataset includes 5 attributes related to the severity (benign or malignant) of a mammographic mass lesion in 830 patients, based on the characteristics of BI-RADS and the patient’s age.

### 3.7. Saheart

The dataset contains information on 462 men living in a high-risk region for coronary heart disease in the Western Cape, South Africa. It is characterized by 9 attributes. The class label indicates whether the person has coronary heart disease: negative (0) or positive (1).

### 3.8. Spectfheart

The dataset contains information on the diagnosis of single proton emission computed tomography (SPECT) images of the heart in 267 patients. Each record is described by 44 attributes, and each patient is classified into one of two categories: normal (0) or abnormal (1).

### 3.9. Wisconsin Diagnosis Breast Cancer (WDBC)

The dataset contains 569 records with 30 features computed from a digitized image of a breast mass. These attributes describe the characteristics of the cell nuclei present in the image. The task is to predict whether the tumor found is benign or malignant.

### 3.10. Wisconsin Breast Cancer Original (Wisconsin)

The dataset contains 9 attributes with 683 cases from a study of patients who had undergone breast cancer surgery. The task is to predict whether the detected tumor is benign (2) or malignant (4).

### 3.11. Complications

The dataset contains 107 cases of perioperative complications of radical hysterectomy in patients with cervical cancer described by 8 attributes. The task is to determine the presence or absence of perioperative complications [13].

### 3.12. Diabetes

Data was collected from 230 schoolchildren between the ages of 6 and 18 under the care of a pediatric diabetes clinic. It contains 9 parameters, including weekly physical activity parameters. The task is to determine the presence or absence of type 1 diabetes [55].

## 4. Fuzzy Rule-Based Classification Algorithms

This section contains descriptions of the classification algorithms used in these experiments. The algorithms implementations, except for GPR, come from KEEL Included Algorithms [53] and belong to the Rule Learning for Classification family. We used a custom implementation of GPR [56], and set the parameters to default values.

### 4.1. One Rule (1R-C)

1R is an algorithm that ranks attributes according to their error rate, with the attribute with the lowest error rate chosen for the decision tree. The range of values for the selected attribute is then divided into several disjoint intervals, with the number of intervals determined by the value of the SMALL parameter. Finally, the algorithm uses these intervals to create a one-level decision tree, which is a tree with a single decision node that classifies objects based on the chosen attribute [15]. The SMALL parameter was set to 6.

### 4.2. C4.5 (C4.5-C)

C4.5-C is probably the most widely used machine learning algorithm for generating a decision tree [16]. It is an extension of Quinlan’s earlier ID3 algorithm [57]. The pruned parameter that determines whether the algorithm will prune the decision tree was set to TRUE. The confidence parameter determines the minimum confidence required for a rule to be considered significant, and in this case it was set to 0.25. The instances per leaf parameter determines the minimum number of instances that must be present at a leaf node and it was set to 2.

### 4.3. C4.5Rules (C45Rules-C)

C45Rules-C is an algorithm that reads the decision tree or trees produced by C4.5 and generates a set of rules for each tree and all trees together [57,58]. The confidence factor, item sets per leaf, and threshold parameters can be adjusted to fine-tune the generated rules for optimal performance. In the current implementation, the confidence factor was set to 0.25, the item sets per leaf parameter was set to 2, and the threshold was set to 10.

### 4.4. C4.5Rules Simulated Annealing Version (C45RulesSA-C)

C45RulesSA-C is a version of the C45Rules-C algorithm with a general-purpose local search method called Simulated Annealing that generates an approximate solution within a range close to the current solution and accepts the approximate solution if the objective function improves [57,58]. The user-defined parameters such as confidence, item sets per leaf, and threshold are used to fine-tune the generated rules, while the max coldings, max trials, mu, phi, and alpha parameters are used to control the behavior of the Simulated Annealing method. In the current implementation, these parameters were set to 0.25, 2, 10, 10, 0.5, 0.5, and 0.5 respectively.

### 4.5. Hybrid Decision Tree-Genetic Algorithm (DT_GA-C)

DT_GA-C is a hybrid decision tree/genetic algorithm method that allows discovering knowledge from data expressed as easy-to-interpret high-level classification rules [20]. A genetic algorithm aims to generate rules covering examples belonging to small disjuncts, whereas a conventional decision tree algorithm aims to produce rules covering examples of large disjuncts. The user-defined parameters of DT_GA-C, such as confidence was set to 0.25, the instances per leaf parameter was set to 2, and the genetic algorithm approach parameter was set to GA-LARGE-SN. The threshold S to consider a small disjunt parameter was set to 10, the number of total generations for the GA parameter was set to 50, and the number of chromosomes in the population parameter was set to 200. Crossover probability was set to 0.8, and the mutation probability parameter was set to 0.01.

### 4.6. Oblique Decision Tree with Evolutionary Learning (DT_Oblique-C)

DT_Oblique-C uses evolutionary algorithms to optimize split criteria during constructing oblique trees [21]. This allows the algorithm to quickly and efficiently find high-quality split criteria that accurately classify the data. In the current implementation, the number of total generations for the genetic algorithm was set to 25, indicating that the algorithm will run for up to 25 generations before stopping.

### 4.7. Exemplar-Aided Constructor of Hyperrectangles (EACH-C)

EACH-C implements the nested generalized exemplar (NGE) theory. It makes predictions and classifications based on examples that it has seen in the past. The algorithm compares new examples with those it has seen before and finds the closest example in memory. Distance measure aims to determine what is closest [17]. The feature adjustment rate was set to 0.2, and the use second chanse parameter was set to TRUE.

### 4.8. Classifier Based on Fuzzy Logic and Gene Expression Programming (GPR)

GPR is an extremely simple classifier that consists of highly interpretable fuzzy metarules [8]. It uses only two fuzzy sets with linear and complementary membership functions for every continuous feature. The number of populations was set to 500, the number of generations was set to 10, threshold was set to 0.5, and the probability of triggering an operation on the chromosome was set to 0.1.

### 4.9. Hierarchical Decision Rules (Hider-C)

Hider-C uses an approach based on evolutionary algorithms to learn rules in continuous and discrete domains. The algorithm produces a hierarchical set of rules. It uses real and binary coding for individuals in the population [23]. The population size, number of generations, mutation probability and cross percent parameters are used to control the behavior of the genetic algorithm component. In this case, these parameters are set to 0.25, 100, 100, 0.5, and 80 respectively. The extreme mutation probability, prune examples factor, penalty factor, and error coefficient parameters are used to fine-tune the generated rules and control the behavior of the decision tree component of DT_Oblique-C. In this case, the extreme mutation probability is set to 0.05, the prune examples factor is set to 0.05, the penalty factor is set to 1, and the error coefficient is set to 0.

### 4.10. New Structural Learning Algorithm in a Vague Environment (NSLV-C)

NSLV-C is an extention of the iterative scheme of SLAVE that aims to improve the efficiency of the learning process by obtaining complete rules in each iteration and reducing the learning time [59]. It modifies the iterative scheme and the genetic algorithm to remove the bias of the class order and find the best rule in each iteration without fixing the class. We set the study parameters in this study as follows: the population size was set to 100, the maximum number of iterations allowed without change was set to 500, the binary mutation probability was set to 0.01, the integer mutation probability was set to 0.01, the real mutation probability was set to 1.0, and the crossover probability was set to 1.0.

### 4.11. Organizational Co-Evolutionary Algorithm for Classification (OCEC-C)

OCEC-C causes the evolution of sets of examples and, finally, extracts rules from these sets at the end of the evolutionary process [24]. Due to the differences between the individuals in traditional evolutionary algorithms and organizations formed from these sets of examples, three evolutionary operators and a selection mechanism have been developed for realizing the evolutionary operations performed on organizations. It prevents evolutionary processes from producing meaningless rules. The number of total generations was set to 500, and the number of migrating/exchanging members was set to 1.0.

### 4.12. Ordered Incremental Genetic Algorithm (OIGA-C)

OIGA-C address incremental training of input attributes for classifiers [25]. OIGA learns input attributes one after another, and the resulting classification rule sets are also incrementally evolved to accommodate the new attributes. The attributes are arranged in different orders when their discriminating abilities are evaluated. The parameters were set as follows: the mutation probability was set to 0.01, the crossover rate was set to 1.0, the population size was set to 200, the number of rules was set to 30, the stagnation limit was set to 30, the generation limit was set to 200, the survivors percent was set to 0.5, and the attribute order was set to descendent.

### 4.13. Pittsburgh Genetic Interval Rule Learning Algorithm (PGIRLA-C)

PGIRLA-C uses genetic algorithms with real genes to evolve the classification rule sets. The rule sets are evolved by genetic algorithms using the Pittsburgh approach [26]. We set the number of generations to 5000, the population size to 61, the crossover probability to 0.7, the mutation probability to 0.5, and the number of rules to 20.

### 4.14. Repeated Incremental Pruning to Produce Error Reduction (Ripper-C)

Ripper-C is a rule-based classification algorithm proposed by Cohen that derives a set of rules from the training set that match or exceed the performance of decision trees [18]. The three stages of RIPPER-C are growing, pruning, and optimizing. The grow_pct parameter was set to 0.66, and k to 2.

### 4.15. Structural Learning Algorithm in a Vague Environment v0 (SLAVEv0-C)

SLAVEv0-C is a classifier based on fuzzy rules that is generated evolutionarily. Fuzzy rules are evolved for each two-class problem using a Michigan iterative learning approach and integrated using the fuzzy round-robin class binarization scheme [22]. The parameters were set as follows: the population size was set to 20, the number of iterations allowed without change was set to 500, the mutation probability was set to 0.5, the crossover probability was set to 0.1, and lambda was set to 0.8.

### 4.16. Structural Learning Algorithm in a Vague Environment 2 (SLAVE2-C)

SLAVE2-C is a modification of the original SLAVE learning algorithm, including new genetic operators to reduce learning time, improve understanding of the rules obtained, and a new way to penalize the rules in the iterative approach that allows the system’s behavior to improve [60]. The following parameters were set: the population size was set to 20, the number of iterations allowed without change was set to 500, the binary mutation probability was set to 0.5, the binary crossover probability was set to 0.1, the real mutation probability was set to 1.0, the real crossover probability was set to 0.2, and lambda was set to 0.8.

## 5. Performance Metrics

The selection of metrics that measure the performance of algorithms is an essential step in machine learning approaches. Each metric has specific characteristics and measures properties that may be different from the predicted results. The metrics used to evaluate the performance of the proposed work are listed below.

Accuracy (ACC) is calculated by dividing the number of correctly classified samples by the total number of samples in the evaluation dataset. If the model’s predictions for a sample exactly match the true labels for that sample, the subset accuracy is 1.0; otherwise, it is 0.0. The fraction of correct predictions over $n_{\mathrm{samples}}$ is calculated using the *accuracy_score* function from the sklearn.metrics module defined as follows:

(1) $$\mathrm{ACC}\left(y,\hat{y}\right)=\frac{1}{n_{\mathrm{samples}\;}}\sum_{i=0}^{n_{\mathrm{samples}\;}-1}1\left({\hat{y}}_{i}=y_{i}\right)$$

where ${\hat{y}}_{i}$ is the predicted value of the *i*-th sample, $y_{i}$ is the true value for that sample, and 1(x) is the indicator function.

Precision (Pre) is calculated as the ratio of correctly classified samples to all samples assigned to a particular class. Pre is the ability of the classifier to not label a negative sample as positive. It is bounded between 0 and 1, where 1 is the best possible value and 0 is the worst possible value. The metrics for each label, and averages weighted by support, are calculated. It is defined by:

(2) $$\mathrm{Pre}=\frac{1}{\sum_{l\in L}\left|y_{l}\right|}\sum_{l\in L}\left|y_{l}\right|P\left(y_{l},{\hat{y}}_{l}\right)$$

where *y* the set of true (sample, label) pairs, $\hat{y}$ the set of predicted (sample, label) pairs, *L* the set of labels, $y_{l}$ the subset of *y* with label *l*, ${\hat{y}}_{s}$ and ${\hat{y}}_{l}$ are subsets of $\hat{y}$, $P\left(A,B\right):=\frac{\left|A\cap B\right|}{\left|B\right|}$ for some sets *A* and *B*. It is calculated using the *precision_score* method from the sklearn.metrics module.

Sensitivity (Sen) (also known as the Recall) is calculated as the ratio between correctly classified positive samples and all samples assigned to the positive class. Sen is the ability of the classifier to correctly classify all positive samples as positive. It is defined as follows:

(3) $$\mathrm{Sen}=\frac{1}{\sum_{l\in L}\left|y_{l}\right|}\sum_{l\in L}\left|y_{l}\right|R\left(y_{l},{\hat{y}}_{l}\right)$$

where *y* the set of true (sample, label) pairs, $\hat{y}$ the set of predicted (sample, label) pairs, *L* the set of labels, $y_{l}$ the subset of *y* with label *l*, ${\hat{y}}_{s}$ and ${\hat{y}}_{l}$ are subsets of $\hat{y}$, $R\left(A,B\right):=\frac{\left|A\cap B\right|}{\left|A\right|}$. It is calculated using the *recall_score* method from the sklearn.metrics module.

Other performance metrics are calculated using the well-known confusion matrix which consists of four entries: the true positives (TP), false negatives (FN), false positives (FP), and true negatives (TN) [61], as follows:

(4) $$M=\left(\begin{array}{cc} \mathrm{TP} & \mathrm{FN}\\ \mathrm{FP} & \mathrm{TN} \end{array}\right)$$

True Positives (TP) refer to the number of samples correctly classified as positive, e.g., the number of records that have breast cancer correctly predicted as having breast cancer. True Negatives (TN) refer to the samples correctly classified as negative, e.g., the number of records without breast cancer correctly predicted to be non-breast cancer. False Positives (FP) refer to the samples incorrectly classified as positive, e.g., the number of samples without breast cancer incorrectly predicted to have breast cancer. False Negatives (FN) refer to the samples incorrectly classified as negative, e.g., the number of records containing breast cancer is incorrectly predicted not to have breast cancer.

Specificity (Spe) is calculated as the ratio between correctly classified negative samples and all samples classified as negative. Spe is bounded to [0, 1], where 1 represents perfect predictions of the negative class and 0 represents incorrect predictions of all samples in the negative class. It is defined by:

(5) $$\mathrm{Spe}=\frac{\mathrm{TN}}{\mathrm{TN}+\mathrm{FP}}$$

Area Under ROC Curve (AUC) measures the ability of a classifier to distinguish between classes and is used to summarize the ROC curves. The higher AUC, the better model’s performance in distinguishing between the positive and negative classes. The ROC curve is plotted with Sen against the false positive rate (FPR, calculated as 1−Spe). Sen is on the *y*-axis, and FPR is on the *x*-axis.

Matthews Correlation Coefficient (MCC) is a correlation coefficient between true and predicted classes. It reaches a high value only if the classifier achieves good results in all entries in the confusion matrix. MCC is bounded to [−1, 1], where 1 represents a perfect prediction, 0 random guessing, and −1 represents total disagreement between prediction and observation [62]. MCC has become popular research applied in machine learning due to its favorable properties in the case of imbalanced classes. It is defined as follows:

(6) $$\mathrm{MCC}=\frac{\mathrm{TP}\times \mathrm{TN}-\mathrm{FP}\times \mathrm{FN}}{\sqrt{\left(\mathrm{TP}+\mathrm{FP})(\mathrm{TP}+\mathrm{FN})(\mathrm{TN}+\mathrm{FP})(\mathrm{TN}+\mathrm{FN}\right)}}$$

Weighted Metric (WM) is a single performance indicator for multiple metrics that was proposed in this study to make it easier to compare algorithms and select the optimal algorithm:

(7) $$\mathrm{WM}=\frac{30\times AUC+50\times Sen+5\times (ACC+Pre+Spe+MCC)}{100}$$

According to some studies [63], the AUC is one of the most significant measures of a classifier’s performance, so that it was included with a weight of 0.3. The Sen term is also often used in health care and medical research to describe the confidence in results and utility of testing. Therefore, it was weighted with 0.5 when calculating WM, and other metrics were weighted with 0.05.

## 6. Experimental Results

A fuzzy rule-based algorithms’ performance is evaluated in this section. Algorithms compared include: AdaBoost.NC-C, CART-C, C45-C, C45Rules-C, C45RulesSA-C, Chi-RW-C, EACH-C, FH-GBML-C, FURIA-C, DT_GA-C, MPLCS-C, DT_Oblique-C, OIGA-C, OCEC-C, 1R-C, and GPR. Table 2 shows the average results of ACC, AUC, Pre, Sen, and Spe obtained on all datasets using 10-fold cross-validation. The results in Table 2 are sorted in descending order based on MCC, and the three best results for each metric are highlighted in bold.

GPR achieved the highest MCC of 0.459 ± 0.342, while 1R-C achieved the lowest MCC of 0.228 ± 0.331. In terms of ACC, OIGA-C (0.860 ± 0.253), PGIRLA-C (0.819 ± 0.269), and GPR (0.807 ± 0.281) obtained the best results. Ripper-C had the highest AUC score of 0.730 ± 0.162, followed by C45RulesSA-C and OCEC-C. The best Spe obtained NSLV-C (0.795 ± 0.122), OIGA-C (0.793 ± 0.114), and GPR (0.792 ± 0.125). OIGA-C achieved the highest Pre of 0.782 ± 0.152. According to Sen, NSLV-C achieved the highest results (0.795 ± 0.122), followed by OIGA-C (0.793 ± 0.114) and GPR (0.792 ± 0.125), and EACH-C achieved the worst performance (0.662 ± 0.185). OIGA-C, GPR, and NSLV-C had the highest WM scores of 0.755 ± 0.138, 0.753 ± 0.145, and 0.752 ± 0.141, respectively. The algorithms with the lowest WM were EACH-C (0.630 ± 0.180), 1R-C (0.645 ± 0.160), and PGIRLA-C (0.681 ± 0.172).

**Figure 1:**
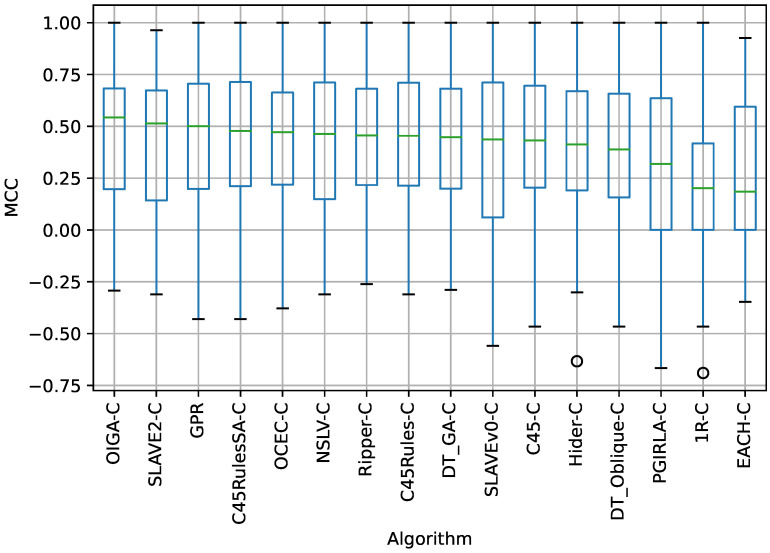
Distribution of the MCC values for each algorithm in all datasets.

The box plot in Figure 1 shows MCC of each algorithm in all datasets subjected to 10-fold cross-validation. The results are sorted in descending order by the median of the MCC. OIGA-C had the highest MCC among all the fuzzy rule-based algorithms tested. SLAVE2-C had the second-highest MCC, while GPR had the third-highest MCC. The plot also shows several outliers that decrease the average results of the algorithms.

**Figure 2:**
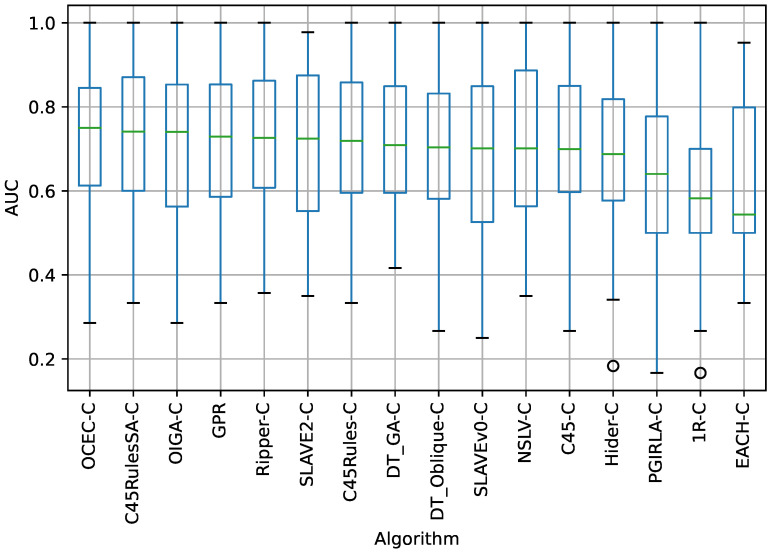
Distribution of the AUC values for each algorithm in all datasets.

The box plot in Figure 2 shows the AUC of each algorithm in all datasets subjected to 10-fold cross-validation. The results are sorted in descending order by the median of the AUC. The best results were obtained by the OCEC-C algorithm, followed by C45RulesSA-C, OIGA-C, GPR, and Ripper-C. The plot also shows several outliers that decrease the average results of the algorithms. The 1R-C and EACH-C algorithms are at the bottom of the list.

**Figure 3:**
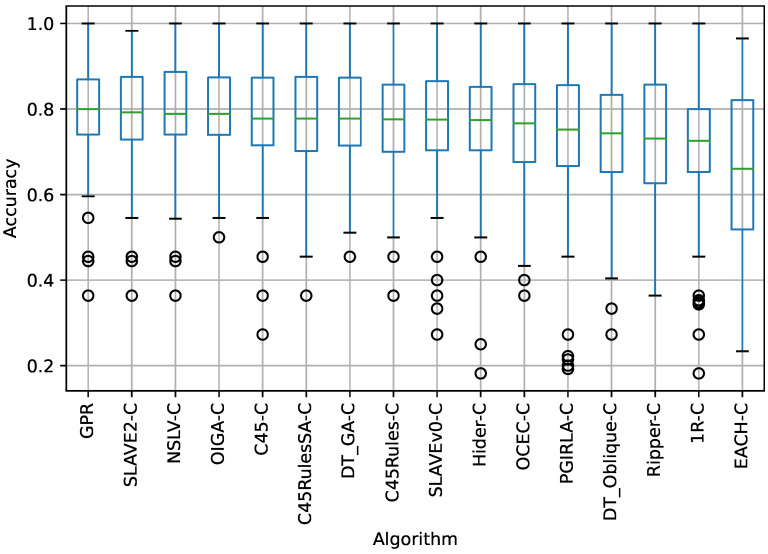
Distribution of ACC values for each algorithm in all datasets.

The box plot in Figure 3 shows the ACC of each algorithm in all datasets subjected to a 10-fold cross-validation. The results are sorted in descending order by the median of the ACC. GPR was found to be the most accurate among all the fuzzy rule-based algorithms tested. Therefore, GPR is a good choice for general use. SLAVE2-C was ranked second and NSLV-C was ranked third. The 1R-C and EACH-C algorithms again took the last two places, similar to their positions in rankings for MCC and AUC.

Table 3 presents the result of comparing the fuzzy rule-based classifier. They are compared in terms of the following metrics, which are calculated as averages for every algorithm in every dataset: ANC—the average number of characters per rule in the dataset, ANR—the average number of rules in the dataset, ANA—the average number of attributes per rule in dataset, ANUA—the average number of unique attributes per rule in dataset. The results are sorted in ascending order by ANC. 1R-C generated an ANC of 106.54 with a small average number of rules on the dataset (ANR of 3.31) and a small average number of attributes on the dataset (ANA of 3.31). However, it achieved the worst results for MCC, AUC, and other performance metrics, as shown in Table 2. The comparison result places GPR near 1R-C also as an algorithm providing an extremely simple and concise set of metarules. Its simplicity is expressed as an ANC of 156.23, which is over 208 times smaller for GPR than for OIGA-C, which achieved the best results in terms of the WM (Table 2). GPR generates an ANR of 4.0 and ANA of 6.69 while maintaining high MCC, ACC, and other performance metrics, as shown in Table 2. DT Oblique-C generated the most complicated rules (ANC of 32457.38, ANA of 1059.08).

Table 4 presents examples of linguistic “if-then” fuzzy rules generated by fuzzy rule-based classifiers on the real Diabetes dataset. The results are sorted alphabetically. Parsing the algorithms’ output files ensured that all the compared rules had the same format. The number of digits in the ranges was not modified and depends on the KEEL implementation. The table also provides information on the number and length of the generated rules. In terms of syntax, GPR generated the shortest and most understandable rules, whereas EACH-C generated the lowest number of rules. OCEC-C generated the largest number of rules, while OIGA-C generated the largest number of characters. The study’s findings suggest that a structure based on four features is at the limit of human processing capacity and such a rule is very hard to understand [64]. Therefore, using algorithms containing several or several dozen attributes is challenging.

The results indicate that GPR generates the shortest and most interpretable rules while still achieving good classification performance. As a result, we decided to use the Wilcoxon signed-rank test to statistically compare the results of GPR with those of other fuzzy rule-based algorithms. Table 5 presents the results of the Wilcoxon signed-rank test. The results of GPR and fuzzy rule-based algorithms for the MCC, AUC, and ACC measurements were compared. *X* denotes a vector containing the mean values of the MCC (or AUC and ACC) measure for the GPR algorithm, as calculated from ten random stratified folds for each dataset. $Y_{i}$ denotes a vector containing the corresponding values for the *i*th algorithm tested on exactly the same folds. The index *i* represents the name of the algorithm, where *i* belongs to the set: {1R-C, C45-C, C45Rules-C, C45RulesSA-C, DT_GA-C, DT_Oblique-C, EACH-C, Hider-C, NSLV-C, OCEC-C, OIGA-C, PGIRLA-C, Ripper-C, SLAVE2-C, SLAVEv0-C}. Table 5 shows the probability (*p*-value) of a two-sided paired Wilcoxon test for the null hypothesis $H_{0}$ that the difference $\left(X-Y_{i}\right)$ follows a distribution with a zero median. The two-sided *p*-value is calculated by doubling the most significant one-sided value.

According to the results in Table 5, for the MCC measure, the Wilcoxon signed-rank test fails to reject the null hypothesis of no significant difference in the mean values of MCC at the significance level of α=0.05 when comparing GPR to the following nine algorithms: C45-C, C45Rules-C, C45RulesSA-C, DT_GA-C, NSLV-C, OCEC-C, OIGA-C, Ripper-C, and SLAVE2-C. However, according to the results in Table 5, the null hypothesis can be rejected at the 5% level when comparing GPR to the following six algorithms: 1R-C, DT_Oblique-C, EACH-C, Hider-C, PGIRLA-C, and SLAVEv0-C. Thus, the alternative hypothesis $H_{1}$ is accepted: there is a significant difference in the mean values of MCC for GPR compared to the 1R-C, DT_Oblique-C, EACH-C, Hider-C, PGIRLA-C, and SLAVEv0-C algorithms. According to Wilcoxon’s rank test (Table 5) and the distribution of MCC values as shown in Figure 1, from the perspective of the MCC criterion, GPR is worse at the significance level of α=0.05 than OIGA-C, and SLAVE2-C. For the same reasons, GPR is better than the following six algorithms: 1R-C, DT_Oblique-C, EACH-C, Hider-C, PGIRLA-C, and SLAVEv0-C.

According to the results in Table 5, for the AUC measure, the Wilcoxon signed-rank test fails to reject the null hypothesis of no significant difference in the mean values of AUC at the significance level of α=0.05 when comparing GPR to the following algorithms: C45-C, C45Rules-C, C45RulesSA-C, DT_GA-C, DT_Oblique-C, NSLV-C, OCEC-C, OIGA-C, Ripper-C, and SLAVE2-C. Considering the *p*-values for the AUC measure in Table 5 and the distribution of AUC values for each algorithm across all datasets and 10 cross-validation folds shown in Figure 2 it can be concluded that GPR is worse than OCEC-C, C45RulesSA-C, and OIGA-C, but better than 1R-C, EACH-C, Hider-C, PGIRLA-C, Ripper-C, and SLAVEv0-C.

According to the results in Table 5, for the ACC measure, the Wilcoxon signed-rank test fails to reject the null hypothesis of no significant difference in the mean values of ACC at the significance level of α=0.05 when comparing GPR to the following algorithms: C45-C, DT_GA-C, NSLV-C, OIGA-C, and SLAVE2-C. Based on the *p*-values for the ACC measure in Table 5 and the distributions of ACC values shown in Figure 3, GPR is superior at the significance level of α=0.05 to the following algorithms: 1R-C, C45Rules-C, C45RulesSA-C, DT_Oblique-C, EACH-C, Hider-C, OCEC-C, PGIRLA-C, Ripper-C, and SLAVEv0-C.

## 7. Discussion and Conclusions

Machine learning can be used to improve the accuracy and objectivity of clinical experts in clinical decision-support systems. Generated rules can help identify the most likely diagnosis and show how individual attributes contributed to the decision. However, it can be difficult to select the most relevant rules from the many that are generated, especially when they contain numerous attributes and are difficult to interpret. It is important to choose the appropriate algorithm for the task at hand to ensure the best results. This paper has proposed a comparative study of fuzzy rule-based algorithms that were applied to issues in the field of clinical decision support. The proposed comparison begins with applying 16 different rule-based fuzzy logic algorithms: 1R-C, C45-C, C45Rules-C, C45RulesSA-C, DT_GA-C, DT_Oblique-C, EACH-C, GPR, Hider-C, NSLV-C, OCEC-C, OIGA-C, PGIRLA-C, Ripper-C, SLAVE2-C, SLAVEv0-C to 12 clinical datasets and generation of rules. We calculated performance metrics such as MCC, ACC, AUC, Spe, Pre, Sen, and WM based on the results obtained and compared them. Based on the WM criterion, which takes into account the results obtained from all metrics, the best algorithms are OIGA-C, GPR, and NSLV-C, and the worst are EACH-C, 1R-C, and PGIRLA-C. Then, we presented the MCC, ACC, and AUC values distribution for each algorithm in all datasets. The average length of the rules in the dataset, the average number of rules in the dataset, and the average number of attributes and unique attributes per rule were also included in the comparison. We also presented rules generated for a Diabetes dataset considering the number of rules, their length, and their syntax. Most interpretable rules were generated by 1R, GPR and C45Rules-C. The longest and most complicated rules were generated by DT_Oblique-C, OIGA-C and OCEC-C. In conclusion, algorithms that achieve high classification results tend to generate very complex and lengthy rules (such as OIGA-C), while algorithms that produce simpler rules often have lower classification results (like 1R-C).

The research indicates that GPR generates the shortest and most interpretable rules while still achieving good classification performance. As a result, we decided to test GPR statistically using the Wilcoxon signed-rank test. It was performed to compare the means of every rule-based fuzzy logic classifier and GPR. According to the results of this test and the distribution of ACC values for each rule-based fuzzy logic algorithm in all datasets, the GPR algorithm outperformed at the significance level of α=0.05 the 1R-C, C45Rules-C, C45RulesSA-C, DT_Oblique-C, EACH-C, Hider-C, OCEC-C, PG1RLA-C, Ripper-C, and SLAVEvO-C algorithms. Considering all the results, we can conclude that GPR can be used successfully for generating rules from medical data.

However, theoretical results, particularly those related to the “no free lunch” theorem [65], state that in the general case no algorithm can outperform every other algorithm in all possible tasks. In other words, there is no one-size-fits-all solution to all problems. The GPR algorithm also has some drawbacks. For example, it uses a genetic algorithm to generate metarules, which can be computationally intensive and slow to converge, especially for large and complex problems. Furthermore, GPR requires the normalization of continuous input data to the interval [0, 1], encoding of all data (continuous and categorical), and the adoption of a threshold for the discriminant function (with a default value of 0.5). The selection of a fitting function for the evolutionary algorithm (such as accuracy or sensitivity) is also required.

This study has a few limitations that should be considered when interpreting the results. First, we did not conduct a memory requirement test or measure run time. Second, we use the default values for the hyperparameters, which could potentially be adjusted to improve performance. Furthermore, the performance of the algorithms was tested only on medical datasets with a relatively small number of records, so the results may not be representative for larger datasets.

One potential area for future research is to conduct further research on the impact of memory requirements and run time. Another idea for future research is to include a greater number of algorithms and real-world datasets obtained through cooperation with various medical organizations. To make our findings more accessible and user-friendly, we also intend to develop a user interface based on our open-source code. This interface will enable medical professionals to easily generate rules for specific medical problems and display them in a unified way, using the most appropriate algorithm for the task at hand. Through these efforts, we hope to enhance the utility and impact of our work in the field of medical decision-making.

## Figures and Tables

**Table 1:** Summary of datasets used in experiments.

	Dataset	Records	Attributes	Classes	Class Imbalance	Source
1	Appendicitis	106	7	2	0.2471	KEEL
2	Breast cancer	277	9	2	0.4133	KEEL
3	Haberman	306	3	2	0.3600	KEEL
4	Heart	270	13	2	0.8000	KEEL
5	Hepatitis	80	19	2	0.1940	KEEL
6	Mammographic	830	5	2	0.9438	KEEL
7	Saheart	462	9	2	0.5298	KEEL
8	Spectfheart	267	44	2	0.2594	KEEL
9	WDBC	569	30	2	0.5938	KEEL
10	Wisconsin	683	9	2	0.5383	KEEL
11	Complications	107	8	2	0.8136	Real
12	Diabetes	230	9	2	1.0000	Real

**Table 2:** Results of a comparison of fuzzy rule-based algorithms.

No.	Algorithm	MCC	ACC	AUC	Spe	Pre	Sen	WM
1	GPR	**0.459 ± 0.342**	**0.807 ± 0.281**	0.720 ± 0.171	**0.792 ± 0.125**	0.772 ± 0.167	**0.792 ± 0.125**	**0.753 ± 0.145**
2	OIGA-C	**0.457 ± 0.337**	**0.860 ± 0.253**	0.714 ± 0.172	**0.793 ± 0.114**	**0.782 ± 0.152**	**0.793 ± 0.114**	**0.755 ± 0.138**
3	Ripper-C	**0.452 ± 0.319**	0.676 ± 0.243	**0.730 ± 0.162**	0.735 ± 0.158	**0.780 ± 0.139**	0.735 ± 0.158	0.718 ± 0.164
4	C45RulesSA-C	0.449 ± 0.343	0.752 ± 0.255	**0.727 ± 0.172**	0.769 ± 0.140	0.776 ± 0.147	0.769 ± 0.140	0.740 ± 0.157
5	OCEC-C	0.447 ± 0.323	0.753 ± 0.221	**0.726 ± 0.164**	0.753 ± 0.145	0.771 ± 0.145	0.753 ± 0.145	0.730 ± 0.156
6	NSLV-C	0.446 ± 0.338	0.791 ± 0.298	0.716 ± 0.171	**0.795 ± 0.122**	0.771 ± 0.148	**0.795 ± 0.122**	**0.752 ± 0.141**
7	C45Rules-C	0.446 ± 0.340	0.738 ± 0.273	0.724 ± 0.173	0.768 ± 0.142	**0.777 ± 0.141**	0.768 ± 0.142	0.737 ± 0.159
8	DT GA-C	0.442 ± 0.329	0.799 ± 0.267	0.712 ± 0.163	0.784 ± 0.116	0.775 ± 0.138	0.784 ± 0.116	0.746 ± 0.135
9	SLAVE2-C	0.438 ± 0.338	0.792 ± 0.296	0.712 ± 0.170	0.786 ± 0.123	0.769 ± 0.148	0.786 ± 0.123	0.746 ± 0.144
10	C45-C	0.438 ± 0.343	0.785 ± 0.264	0.710 ± 0.171	0.782 ± 0.128	0.772 ± 0.146	0.782 ± 0.128	0.743 ± 0.147
11	Hider-C	0.414 ± 0.336	0.797 ± 0.274	0.693 ± 0.167	0.767 ± 0.138	0.763 ± 0.144	0.767 ± 0.138	0.728 ± 0.150
12	DT Oblique-C	0.402 ± 0.346	0.741 ± 0.222	0.703 ± 0.173	0.745 ± 0.146	0.754 ± 0.149	0.745 ± 0.146	0.715 ± 0.160
13	SLAVEv0-C	0.394 ± 0.374	0.761 ± 0.315	0.691 ± 0.182	0.772 ± 0.137	0.749 ± 0.161	0.772 ± 0.137	0.727 ± 0.158
14	PGIRLA-C	0.327 ± 0.337	**0.819 ± 0.269**	0.655 ± 0.165	0.716 ± 0.193	0.668 ± 0.239	0.716 ± 0.193	0.681 ± 0.172
15	EACH-C	0.264 ± 0.340	0.621 ± 0.417	0.626 ± 0.165	0.662 ± 0.185	0.675 ± 0.238	0.662 ± 0.185	0.630 ± 0.180
16	1R-C	0.228 ± 0.331	0.652 ± 0.378	0.610 ± 0.160	0.703 ± 0.162	0.636 ± 0.211	0.703 ± 0.162	0.645 ± 0.160

**Table 3:** Comparison of fuzzy rule-based classifiers in terms of rules’ size metrics.

	Algorithm	ANC	ANR	ANA	ANUA
1	1R-C	106.54	3.31	3.31	1.00
2	GPR	156.23	4.00	6.69	5.31
3	C45Rules-C	392.08	8.38	18.85	6.46
4	C45RulesSA-C	557.62	9.77	28.08	6.15
5	EACH-C	695.384	2.00	23.46	11.85
6	NSLV-C	824.08	8.92	28.46	8.23
7	Ripper-C	981.31	16.15	51.31	8.85
8	C45-C	1425.31	11.46	57.23	6.62
9	DT GA-C	2703.38	18.08	123.00	10.38
10	SLAVE2-C	4593.85	12.38	154.62	13.31
11	SLAVEv0-C	5101.92	14.69	168.08	13.38
12	PGIRLA-C	6330.54	18.69	115.31	12.31
13	Hider-C	11,468.85	18.08	425.15	11.08
14	OCEC-C	12,188.08	83.23	772.46	13.31
15	OIGA-C	20,958.08	30.00	399.23	15.85
16	DT Oblique-C	32,457.38	61.15	1059.08	11.69

**Table 4:** Example of “if-then” fuzzy rules generated by fuzzy rule-based classifiers on the real Diabetes dataset.

Algorithm	Rules Generated for the Diabetes Dataset	Number of Rules	Rules Length
1R-C	IF step count = [13072.0, 55333.0) THEN 0 IF step count = [55333.0, 58288.0) THEN 1 IF step count = [58288.0, 60294.0) THEN 0 IF step count = [60294.0, 114655.0] THEN 1	4	172
C45-C	IF step count <= 60837.000000 AND vigorious <= 128.750000 AND weight <= 80.500000 THEN 0 IF step count <= 60837.000000 AND vigorious <= 128.750000 AND weight > 80.500000 THEN …	12	1828
C45Rules-C	IF height>1.61 AND age>14.0 AND weight<=52.0 THEN 1 IF vigorious>128.75 AND vigorious<=319.5 AND age>8.0 AND moderate>214.916666666667 THEN 1 IF step count>60837.0 THEN 1 …	8	400
C45RulesSA-C	IF height>1.61 AND age>14.0 AND weight<=52.0 THEN 1 IF vigorious>128.75 AND vigorious<=319.5 AND age>8.0 AND moderate>214.916666666667 THEN 1 IF step count>60837.0 THEN 1 …	8	400
DT GA-C	IF step count <= 60837.0 AND vigorious <= 128.75 AND weight <= 80.5 THEN 0 IF step count <= 60837.0 AND vigorious <= 128.75 AND weight > 80.5 THEN 1 IF step count <= 60837.0 …	19	2856
DT Oblique-C	IF -1.0*step count + 60837.0 >= 0 AND -1.0*vigorious + 128.75 >= 0 AND -1.0*weight + 80.5 >= 0 AND -1.0*height + 1.87 >= 0 AND 168.486174002403*sex + -178.36864022034422*age + - …	30	8625
EACH-C	IF age in [6.0, 18.0] AND weight in [19.3, 98.8] AND height in [1.15, 1.88] AND step count in [13072.0, 60837.0] AND sedentary in [1343.16666666667, 7813.33333333333] AND l …	2	603
GPR	IF step count is High THEN 1 IF vigorious is High AND sedentary is High THEN 1 ELSE 0	3	87
Hider-C	IF age = [7.5, 17.5) AND weight = [29.15, 65.7) AND step count = [ , 55096.5) AND sedentary = [2270.083333333335, 4964.916666666664) AND light = [356.875, 1330.833333333335) AND …	14	3595
NSLV-C	IF step count = { VeryLow Low} THEN 0 IF step count = { High VeryHigh} THEN 1 IF age = { Low High VeryHigh} AND moderate = { Low VeryHigh} THEN 1	3	145
OCEC-C	IF step count = 3 THEN 1 IF age = 2 AND sedentary = 1 THEN 1 IF sex = 0 AND vigorious = 1 THEN 1 IF sex = 0 AND step count = 1 AND light = 1 THEN 0 IF height = 2 AND ste …	62	6763
OIGA-C	IF 1.6699878586619132 < sex < 1.1982191470913168 AND 9.4429624945491 < age < 16.56761035848586 AND 67.72250192298611 < weight < 85.23233850170679 AND 1.859257826523217 < height …	30	14312
PGIRLA-C	IF sedentary = [3801.8675692824663, 5006.615988626676] AND light = [1162.1170959360238, 2362.4439084883884] AND moderate = [414.0390532025578, 474.55751714327096] AND vigorious …	19	4340
Ripper-C	IF step count<=60837.0 AND height<=1.58 THEN 0 IF step count<=60837.0 AND moderate<=119.0 THEN 0 IF step count<=60837.0 AND vigorious<=127.5 AND height>1.64 AND moderate>123 …	9	467
SLAVE2-C	IF age = { VeryLow Medium} AND weight = { Medium} AND height = { High VeryHigh} AND step count = { VeryLow Low} AND sedentary = { Medium} AND light = { Low} AND moderate = { Low …	8	2098
SLAVEv0-C	IF step count = { VeryLow Low} THEN 0 IF age = { VeryLow Low Medium VeryHigh} AND height = { VeryLow Low Medium VeryHigh} AND step count = { Medium} AND sedentary = { Medium} …	11	2814

**Table 5:** Comparison of fuzzy rule-based classifiers and GPR with Wilcoxon’s signed-rank test.

No.	Algorithm	MCC *p*-Value	AUC *p*-Value	ACC *p*-Value
1	1R-C	0.0000	0.0000	0.0000
2	C45-C	0.8475	0.6052	0.2622
3	C45Rules-C	0.8690	0.0899	0.0027
4	C45RulesSA-C	0.6243	0.0583	0.0123
5	DT GA-C	0.9265	0.6479	0.3322
6	DT Oblique-C	0.0026	0.0592	0.0000
7	EACH-C	0.0000	0.0000	0.0000
8	Hider-C	0.0056	0.0016	0.0022
9	NSLV-C	0.5980	0.8152	0.7802
10	OCEC-C	0.0725	0.8430	0.0000
11	OIGA-C	0.6399	0.3130	0.8192
12	PGIRLA-C	0.0003	0.0004	0.0001
13	Ripper-C	0.5355	0.4273	0.0000
14	SLAVE2-C	0.2653	0.2346	0.4621
15	SLAVEv0-C	0.0012	0.0014	0.0023

## Data Availability

All reported results can be found at https://github.com/czmilanna/rules, accessed on 12 January 2023.

## Abbreviations

The following abbreviations are used in this manuscript:

ACC	accuracy
ANA	average number of attributes per rule in dataset
ANC	average number of characters per rule in dataset
ANR	average number of rules on dataset
ANUA	average number of unique attributes per rule in dataset
AUC	area under ROC curve
C45RulesSA-C	C4.5Rules simulated annealing version
DT_GA-C	hybrid decision tree-genetic algorithm
DT_Oblique-C	oblique decision tree with evolutionary learning
EACH-C	exemplar-aided constructor of hyperrectangles
FN	false negatives
FP	false positives
FPR	false positive rate
FRBS	fuzzy rule-based systems
GPR	classifier based on fuzzy logic and gene expression programming
Hider-C	hierarchical decision rules
KNN	k-nearest neighbors
MCC	Matthews correlation coefficient
MDSS	medical decision support systems
NSLV-C	New SLAVE
OCEC-C	organizational co-evolutionary algorithm for classification
OIGA-C	ordered incremental genetic algorithm
PGIRLA-C	Pittsburgh genetic interval rule learning algorithm
Pre	precision
RBS	rule-based systems
Ripper-C	repeated incremental pruning to produce error reduction
Sen	sensitivity
SLAVEv0-C	structural learning algorithm in a vague environment
SVM	support vector machine
TN	true negatives
TP	true positives
WDBC	Wisconsin diagnosis breast cancer
WM	weighted metric
Wisconsin	Wisconsin breast cancer (original)
